# Racial Differences of Attitudes Towards Medical Marijuana Use Among American Older Adults

**DOI:** 10.1093/geroni/igaa057.3382

**Published:** 2020-12-16

**Authors:** Kedong Ding, Yifan Lou

**Affiliations:** 1 University of Chicago School of Social Service Administration, Chicago, Illinois, United States; 2 Columbia University, New York, New York, United States

## Abstract

Background: Extensive evidence documented that medical marijuana (MM) could be used for pain management with fewer side effects. Although MM legalization remains controversial as it is perceived to be a “gateway drug”, more states are striving for MM legalization and calling for health insurance coverage. Racial difference in the use of opioids for pain management is well-documented, which may also exist in the use of MM. This study explored an understudied topic on how people from different racial backgrounds perceive MM and its behind mechanism. Method: Data is from Health and Retirement Study Wave 2018 who answered the special modules on MM (n=1340). The attitude is proxied by two dichotomous measures on whether they think MM is acceptable and would lead to hard drug. Logistic regressions were used to evaluate the relationships between attitude and race, adjusted for sociodemographic, health and mental health, and MM knowledge. Moderating effects of diseases and socioeconomic-status (SES) were tested using interaction terms. Results: Older adults who hold positive attitudes towards MM are more likely to be younger, high SES, using opioids. Hispanics and Blacks are more negative towards MM relative to White counterparts. Blacks with cancer (OR=.30) are less likely to believe MM is acceptable, whereas blacks with arthritis are more likely to accept MM (OR=2.08) compared to Whites. Hispanic females (OR=.26) are more likely to oppose MM while Hispanics with higher education background (OR=4.3) are more likely to hold positive attitudes. Implication: The results may guide development of future guidelines on MM prescription.

